# Historical Occurrence and Composition of Novel Brominated Flame Retardants and Dechlorane Plus in Sediments from an Electronic Waste Recycling Site in South China

**DOI:** 10.3390/toxics12010084

**Published:** 2024-01-18

**Authors:** Chenchen Huang, Yanhong Zeng, Yin-E Liu, Yanting Zhang, Jian Guo, Xiaojun Luo, Bixian Mai

**Affiliations:** 1 School of Environmental Science & Spatial Informatics, China University of Mining & Technology, Xuzhou 221116, China; 2 State Key Laboratory of Organic Geochemistry and Guangdong Provincial Key Laboratory of Environmental Protection and Resources Utilization, Guangzhou Institute of Geochemistry, Chinese Academy of Sciences, Guangzhou 510640, China; 3 Guangdong-Hong Kong-MaCao Joint Laboratory for Environmental Pollution and Control, Guangzhou Institute of Geochemistry, Chinese Academy of Sciences, Guangzhou 510640, China; 4 Guangdong Provincial Key Laboratory of Environmental Protection and Resources Utilization, Guangzhou 510640, China; 5 Guangdong University of Petrochemical Technology, Maoming 525000, China

**Keywords:** novel brominated flame retardants, dechlorane plus, e-waste recycling site, historical occurrence, composition profile

## Abstract

Novel brominated flame retardants (NBFRs) and dechlorane plus (DP) have been widely used as alternatives to traditional BFRs. However, little is known about the temporal trends of NBFR and DP pollution in e-waste recycling sites. In the current study, three composite sediment cores were collected from an e-waste-polluted pond located in a typical e-waste recycling site in South China to investigate the historical occurrence and composition of NBFRs and DP. The NBFRs and DP were detected in all layers of the sediment cores with concentration ranges of 5.71~180,895 and 4.95~109,847 ng/g dw, respectively. Except for 2,3,5,6-tetrabromo-*p*-xylene (*p*TBX) and 2,3,4,5,6-pentabromoethylbenzene (PBEB), all the NBFR compounds and DP showed a clear increasing trend from the bottom to top layers. These results implied the long-term and severe contamination of NBFRs and DP. Decabromodiphenyl ethane (DBDPE) was the most abundant NBFR with the contribution proportions of 58 ± 15%, 73 ± 15%, and 71 ± 18% in three sediment cores, followed by 1,2-bis(2,4,6-tribromophenoxy) ethane (BTBPE) and pentabromobenzene (HBB). The ratios of BTBPE/Octa-BDEs and DBDPE/Deca-BDEs varied from 0.12 to 60 and from 0.03 to 0.49, respectively, which had no clear increase trends with a decrease in sediment depth. As for DP, the *f_anti_* values (the concentration ratios of *anti*-DP to the sum of *anti*-DP and *syn*-DP) in sediment cores ranged from 0.41 to 0.83, almost falling in the range of those in DP technical products, suggesting that DP degradation did not occur in sediment cores. The environmental burdens of DBDPE, BTBPE, HBB, PBT, PBEB, *p*TBX, and DP were estimated to be 34.0, 5.67, 10.1, 0.02, 0.02, 0.01, and 34.8 kg, respectively. This work provides the first insight into the historical contamination status of NBFRs and DP in the sediments of an e-waste recycling site.

## 1. Introduction

Brominated flame retardants (BFRs) have been widely added to various commercial products, including electronic equipment, plastics, textiles, building materials, etc., to increase their fire resistance [1]. However, several widely used BFRs have been gradually listed as persistent organic pollutants by the Stockholm Convention to prohibit their production and use due to their bioaccumulation, biotoxicity, and persistence in the environment, including polybrominated diphenyl ethers (PBDEs), hexabromocyclododecanes, polybrominated biphenyls, etc., collectively referred to as traditional BFRs [2]. As a result, some novel BFRs (NBFRs) have been generated as alternatives and introduced into the market, such as decabromodiphenyl ethane (DBDPE), hexabromobenzene (HBB), bis(2,4,6-tribromophenoxy) ethane (BTBPE), 2,3,4,5,6-pentabromotoluene (PBT), 2,3,5,6-tetrabromo-p-xylene (*p*TBX), and 2,3,4,5,6-pentabromoethylbenzene (PBEB) [1]. According to the statistics, the annual global production of NBFRs has reached 100~180 kilotons [3]. Additionally, dechlorane plus (DP) is also used as an alternative to traditional BFRs with an annual global production of about 6000 megagrams [4]. Moreover, NBFRs and DP are incorporated into commercial products without covalent binding, and as a result, they can easily leach into the environment throughout their industrial production, use, and disposal. As mounting studies show, NBFRs and DP have been broadly detected in various biological and abiotic media from all over the world, posing a severe threat to ecology and human health [1,4].

It is worth mentioning that electronic waste (e-waste) recycling has been identified as one of the most important sources of organic pollutants due to outdated disassembly techniques, such as crushing, acid washing, and open incineration [5]. According to a report, the annual global productions of e-waste were 44.7 and 53.6 million megagrams in 2016 and 2019, respectively, and will increase to 74.7 million megagrams in 2030 [6,7]. Before China’s import ban on e-waste in 2017, over half of the e-waste around the world was transported into China for nearly 30 years, which was mainly dismantled in Guiyu, Taizhou, and Qingyuan [8]. Undoubtedly, these e-waste recycling sites have been the most polluted areas by these pollutants. For example, Ling et al., reported that the concentrations of PBDEs, DBDPE, BTBPE, and HBB in sediments from Guiyu were up to 410,000 ng/g dry weight (dw), 35,000 ng/g dw, 36,000 ng/g dw, and 1530 ng/g dw, respectively [9]; Wu et al., detected elevated levels of PBDEs (7060~17,900 ng/g lipid weight [lw]), DP (125~2360 ng/g lw), DBDPE (13.8~1100 ng/g lw), and BTBPE (2.1~810 ng/g lw) in mud carp collected from an e-waste polluted pond in Qingyuan [10]. However, to date, most studies on NBFR and DP pollution in e-waste recycling sites have been time-point-specific [7,8,11,12]. Little is known about the temporal trends of NBFR and DP pollution in e-waste recycling sites. Considering that these pollutants are hydrophobic with octane–water partition coefficients greater than four, they tend to accumulate in sediments and have almost no migration after deposition [1,13]. Furthermore, the bioavailability of these pollutants in sediments is very low, resulting in their resistance to microbial degradation [14]. Therefore, the sediment cores could function as a reliable record to reconstruct the historical pollution of NBFRs and DP [15,16].

In the present study, three composite sediment cores were collected from an e-waste-polluted pond in a typical e-waste recycling site in South China. The concentrations of six NBFRs (including DBDPE, BTBPE, HBB, PBEB, PBT, and *p*TBX) and DP in these sediment cores were determined to trace their historical occurrence and composition profiles caused by the dismantlement of e-waste. Moreover, the mass inventories of the NBFRs and DP were also calculated to assess their environmental burdens historically. These results are expected to provide a microcosm of the pollution history of NBFRs and DP in e-waste recycling sites around the world.

## 2. Material and Methods

### 2.1. Study Region and Sample Collection

The sampling region is located in the town of Longtang, Qingyuan City, Guangdong Province (23°36′ N, 113°04′ E), which initiated e-waste recycling activities in the 1990s, and was one of the three largest e-waste recycling sites in China. There were more than 1300 e-waste dismantling workshops, with the annual volume of e-waste dismantled being about 1.7 million megagrams [17]. Although crude e-waste recycling activities have been restricted since the early 2010s and family-run e-waste dismantling workshops were finally banned in 2015 by the local government, elevated levels of e-waste-derived pollutants have been often detected in this region to date [12].

Three composite sediment cores, called cores 1, 2, and 3, respectively, were collected at depths of 41 cm from a pond (with an area of 5000 m^2^) near several e-waste-dismantling workshops in 2016. The detailed sampling locations were given in our previous paper [18]. For each composite sediment core, five sediment cores sampled within a 1 m radius were obtained with a Plexiglas gravity corer. These were then sliced into 3 cm intervals in situ, except for the surface layer with 5 cm intervals. All sediment samples were cooled to −2 °C for transportation to the laboratory and preserved at −20 °C until further analysis.

### 2.2. Sample Preparation and Instrumental Analysis

After freeze drying, pulverization, and homogenization, approximately 0.5 g (dw) sediment was Soxhlet-extracted with 200 mL mixed solvent (acetone [Ace]/*n*-hexane [Hex] = 1:1, *v*/*v*). Before the extraction, the surrogate standards (BDE77, BDE181, BDE205, and ^13^C_12_-labeled-BDE209) were spiked into the extraction agent to monitor the recoveries of targets and the activated copper sheets were added for sulfur elimination. The extracts were evaporated to approximately 1 mL by a rotary evaporator, the solvent was exchanged with Hex, and the extracts were purified on a multilayer silica gel column (i.d. = 1.0 cm) containing 8 cm neutral silica (3% H_2_O deactivated, *w*/*w*) and 16 cm sulfuric acid silica (44% sulfuric acid, *w*/*w*) from bottom to top. Then, the targets were eluted with 45 mL of Hex/dichloromethane (1:1, *v*/*v*), which was concentrated to near-dryness using a gentle stream of nitrogen, re-dissolved in 300 µL isooctane, and spiked with internal standards (BDE118, BDE128, 4′-F-BDE67, 3-F-BDE153) before instrumental analysis.

All the NBFRs and DP were analyzed using an Agilent gas chromatograph–mass spectrometer system (6890N GC-5975B MS) with an electron-capture negative chemical ionization (ENCI) ion source. While the DPDPE and BTBPE were separated on a DB-XLB column (15 m × 0.25 mm i.d. × 0.25 μm film thickness; Agilent J&W Scientific, Santa Clara, CA, USA), the remaining NBFRs and DP were determined on the same capillary column with a length of 30 m. More details on the instrumental settings have been described in our previous paper [19]. Ion pairs (*m*/*z*) of 79/81, 653.8/655/8, 618/620, and 584/586 were monitored for NBFRs, *syn*-/*anti*-DP, *anti*-Cl_11_-DP, and *anti*-Cl_10_-DP, respectively.

### 2.3. Quality Assurance and Quality Control (QA/QC)

The procedural blanks without sediments were performed to monitor background pollution levels from the pretreatment process. Because no targets were detected in the blank samples, the method detection limits (MDLs) were defined as ten times the signal-to-noise ratio, calculated from 0.01 to 0.25 ng/g dw. The recovery (mean ± standard deviation [AV ± SD]) of the surrogate standards ranged from 93% to 98%. All the concentration data were not recovery-corrected.

### 2.4. Statistical Analysis

All the statistical analysis and graph drawing were carried out using SPSS 21.0 and Origin 8.0 for Windows. The data were first tested for normality using the One-Sample Kolmogorov–Smirnov test, and all these data were not normally distributed. Therefore, non-parametric tests, including Spearman’s correlation analysis and the Kolmogorov–Smirnov Z test, were employed to analyze the relationship among the data. The level of significance was set at *p* < 0.05 for all statistical analysis.

## 3. Results and Discussion

### 3.1. Contamination Levels of NBFRs and DPs in Sediment Cores

In the current study, a total of six NBFRs (DBDPE, BTBPE, HBB, PBEB, PBT, and *p*TBX) and four DPs (*syn*-DP, *anti*-DP, *anti*-Cl_11_-DP, and *anti*-Cl_10_-DP) were determined in sediment cores sampled from an e-waste recycling site in South China. Their concentrations and detection frequencies have been summarized in Table 1. Except for PBEB, *p*TBX, *anti*-Cl_11_-DP, and *anti*-Cl_10_-DP, with detection frequencies of 23%, 36%, 77%, and 97%, respectively, all the remaining NBFRs and DP compounds were detected in all depth layers of the three sediment cores (Table 1). Moreover, it is recorded that e-waste recycling activities were initiated in the 1990s and banned in 2015 in this region [10]. These results suggested that this region bears long-term contamination of NBFRs and DP. Not only that, but the total concentrations of the six NBFRs (∑_6_NBFRs) in the sediment cores varied from 5.71 to 180,895 ng/g dw (AV ± SD: 14,152 ± 32,871 ng/g dw; median: 960 ng/g dw), whereas those of the four DP compounds (∑_4_DPs) ranged from 4.95 to 109,847 ng/g dw (AV ± SD: 9713 ± 23,677 ng/g dw; median: 920 ng/g dw) (Table 1). On the one hand, the concentrations of NBFRs and DPs in sediments from this region were comparable to or even much higher than those of some other e-waste recycling sites, such as Guiyu (the range of NBFRs: 0.58~73,100 ng/g dw; the range of DPs: 1100~7200 ng/g dw) [9,20] and Taizhou (the range of NBFRs: not detected [n.d.]~7100 ng/g dw; the range of DPs: 0.10~83 ng/g dw) [8,21], another two typical e-waste recycling sites in China, and Bui Dau, Hung Yen Province (the range of NBFRs: 3.1~728 ng/g dw; the range of DPs: 0.97~70 ng/g dw) [22]—an e-waste recycling site in northern Vietnam. Moreover, the concentrations of NBFRs and DPs in sediments from this region were also much higher than those of highly industrial manufacturing areas around the world—another primary source of NBFRs in aquatic environments, such as Lake Shihwa in Korea (the range of NBFRs: n.d.~60 ng/g dw; the range of DPs: n.d.~2.64 ng/g dw) [23] and the Pearl River Delta in South China (the range of NBFRs: 1.52~1785 ng/g dw; the range of DPs: n.d.~9.46 ng/g dw) [24]. On the other hand, elevated NBFR and DP levels have been widely detected in a variety of biological samples in this region, including mud carp (the range of NBFRs: 53.9~1930 ng/g lw; the range of DPs: 125~2360 ng/g lw) [10], water snakes (the range of NBFRs: 166~5100 ng/g lw; the range of DPs: 77~610 ng/g lw) [25], dragonfly larvae (the mean of NBFRs: 28,000 ± 4850 ng/g lw; the range of DPs: 700~5000 ng/g lw) [26,27], and even human hair (the range of NBFRs: 16.4~991 ng/g dw; the range of DPs: 1.64~360 ng/g dw) [28]. These results implied the severe contamination of NBFRs and DPs in the studied region, which could pose a threat to local ecological health.

Additionally, the concentrations (AV ± SD) of both ∑_6_NBFRs (33,271 ± 54,866 ng/g dw) and ∑_4_DPs (22,732 ± 37,997 ng/g dw) in core 3 were significantly higher than those in cores 1 (∑_6_NBFRs: 2455 ± 4617 ng/g dw; ∑_4_DPs: 4506 ± 6582 ng/g dw) and 2 (∑_6_NBFRs: 6631 ± 17,118 ng/g dw; ∑_4_DPs: 1899 ± 3001 ng/g dw) (Kolmogorov–Smirnov Z test: *p* < 0.05) (Table 1). The concentration differences in NBFRs and DP among the three sediment cores could be related to the total organic carbon contents (TOCs) in sediments because of the higher TOCs (AV ± SD) in core 3 (71.1 ± 65.5 mg/g) than those in cores 1 and 2 (29.5 ± 30.0 and 19.9 ± 21.5 mg/g, respectively) [18] and the significantly positive correlations of TOCs with the concentrations of ∑_6_NBFRs (Spearman: *r* = 0.88; *p* < 0.05) and ∑_4_DPs (Spearman: *r* = 0.94; *p* < 0.05).

**Figure 1:**
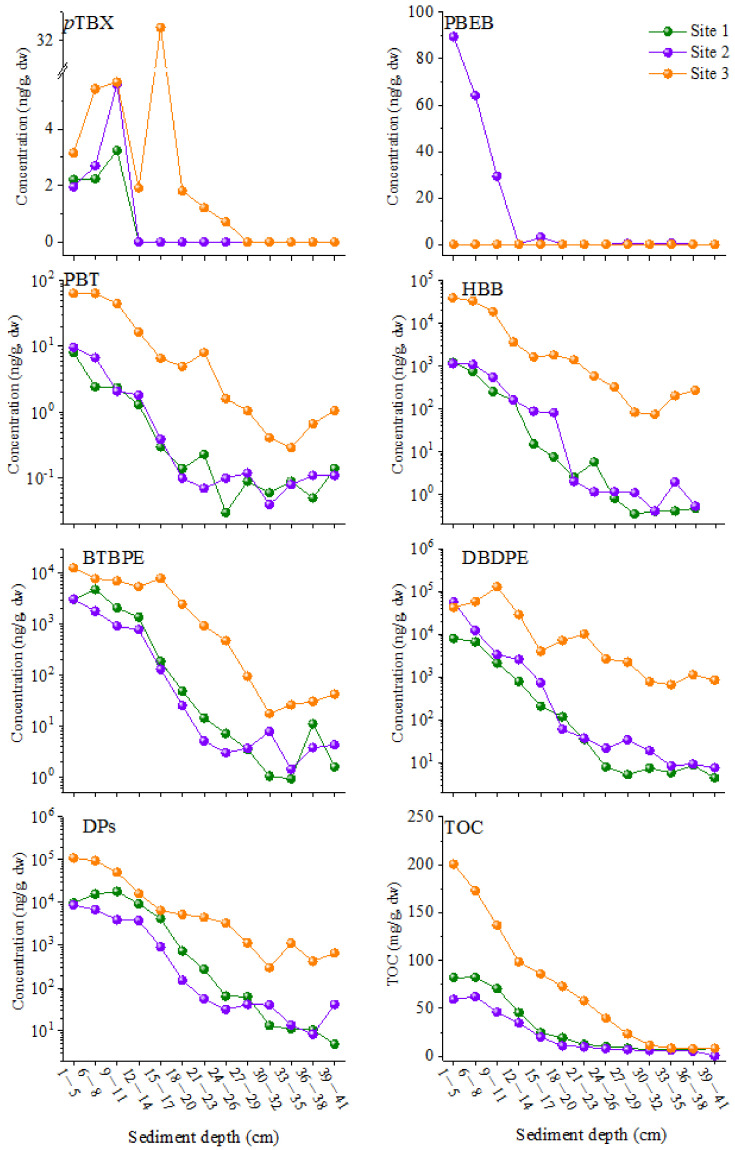
The variation in total organic carbon contents (TOCs) and NBFR and DP levels in the three sediment cores (DP represents the total concentrations of *syn*-DP, *anti*-DP, *anti*-Cl_11_-DP, and *anti*-Cl_10_-DP; the TOC data are cited from our previous paper [18]).

As depicted in Figure 1, except for PBEB and *p*TBX with the lowest concentrations and detection frequencies, all the NBFR and DP compounds’ concentrations generally increased from the bottom to the top layers of the sediment cores, with their levels in surface sediments being 3~4 orders of magnitude higher than those in the bottom layers. For example, the concentrations of DBDPE increased from 4.41 to 8014 ng/g dw, from 7.58 to 56,947 ng/g dw, and from 664 to 128,990 ng/g dw in sediment cores 1, 2, and 3, respectively, while the concentrations of BTBPE increased from 0.93 to 4750 ng/g dw, from 1.44 to 3087 ng/g dw, and from 17.7 to 12,678 ng/g dw in the corresponding sediment cores (Table 1). Furthermore, Spearman’s correlation analysis found that the concentrations of NBFR and DP compounds in sediment cores showed significant correlations with each other (*r* = 0.90~0.97; *p* < 0.05), demonstrating that they originated from a similar source—e-waste dismantlement. To our knowledge, this is the first report on the vertical trends of NBFR and DP concentrations in sediment cores from e-waste recycling sites. The concentration variations in NBFRs and DP in sediment cores were consistent with the increasing amount of e-waste produced worldwide. According to the statistics, the global average generation of e-waste was approximately 20 million megagrams in 2005 and 48.9 million megagrams in 2012, and will increase to more than 75 million megagrams by 2030, with the annual e-waste production rate rising by about 3~5% [29,30]. Before the import ban on e-waste enacted by China in 2017, over half of the global e-waste was transported into China and dismantled primarily in Qingyuan, Guiyu, and Taizhou [8]. In addition, increases in the concentrations of NBFRs and DP were also observed in sediment cores sampled from main industrial and manufacturing areas around the world, such as Lake Shihwa in Korea [23] and the Pearl River Delta in South China [15], demonstrating the increasing production and use of these FRs, which could also contribute to the increase trends of these pollutants in e-waste recycling sites.

### 3.2. Composition Profiles of NBFRs and DP in Sediment Cores

**Figure 2:**
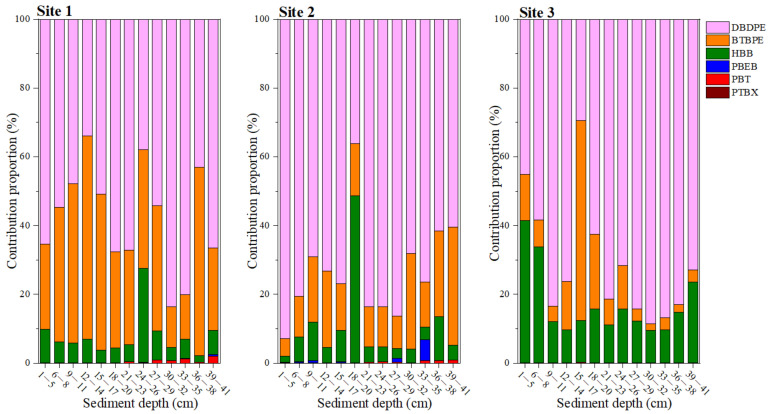
The composition profiles of NBFRs in the three sediment cores.

As shown in Figure 2, DBDPE was the predominant NBFR in sediment cores, with its contribution proportions being up to 58 ± 15%, 73 ± 15%, and 71 ± 18% in the corresponding sediment cores. Additionally, BTBPE and HBB were another two main NBFRs in sediment cores. The contribution proportions of BTBPE were 34 ± 15%, 17 ± 8%, and 12 ± 15% in sediment cores 1, 2, and 3, respectively, whereas HBB accounted for 7 ± 6%, 9 ± 12%, and 17 ± 10% of ∑_6_NBFRs in corresponding sediment cores. As for the remaining three NBFRs (PBEB, PBT, and *p*TBX), they were minor NBFRs in sediment cores, with their total contribution proportions ranging from 0.03% to 6.79%. Similarly, the composition profiles of NBFRs in studied sediment cores were consistent with those of DBDPE, BTBPE, HBB, PBT, and PBEB in sediments from another two primary e-waste recycling sites in China, with average contributions of 71%, 16%, 12%, 0.66%, and 0.01%, respectively, in Taizhou and of 68%, 28%, 3%, 0.16%, and 0.01%, respectively, in Guiyu [8,9]. In addition, DPDPE, BTBPE, and HBB were generally determined to be the three dominant NBFRs in sediments of rivers, lakes, wetlands, and seas from all over the world, such as the highly industrialized saltwater lake in Korea [23], the Pearl River Estuary in South China [15], and the Challenger Deep in the Mariana, Mussau, and New Britain trenches [31]. The composition profiles of NBFRs in sediments could be related to their global production and usage volume. The global production volume of DBDPE was 4540~22,700 megagrams in 2006 and increased to 22,700~45,400 megagrams in 2012 [32], while the global production volume of BTBPE was estimated to be 5000 megagrams in 1997 and increased to >16,000 megagrams in 2001 [33,34]. Additionally, the annual production volumes of HBB, PBEB, and PBT were 350 megagrams in 2001, 4.54~227 megagrams in 1986, and 1000~5000 megagrams in 1997 [35,36].

**Figure 3:**
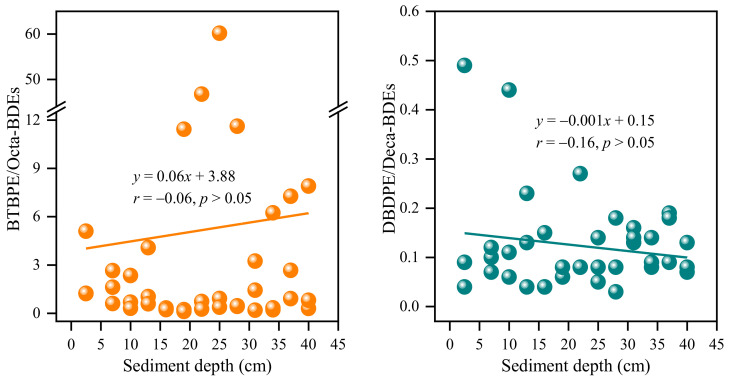
The variation in the ratios of BTBPE to Octa-BDEs and DBDPE to Deca-BDEs (BTBPE/Octa-BDEs and DBDPE/Deca-BDEs) in sediment cores.

Of these, DBDPE and BTBPE were mainly used as alternatives to Deca-BDEs and Octa-BDEs, respectively [35]. Our previous study reported the concentration variations of PBDEs in the same sediment cores, with the concentrations ranging from 65 to 1,030,000 ng/g dw [18]. The concentration ratios of BTBPE to Octa-BDEs (BTBPE/Octa-BDEs) and DBDPE to Deca-BDEs (DBDPE/Deca-BDEs) could be used to trace the replacement rates of NBFRs compared to traditional BFRs. Because Deca-BDEs mainly consist of BDE209 and Octa-BDEs are dominated by BDE153, 183, 196, and 197, the concentrations of these congeners were used to calculate BTBPE/Octa-BDEs and DBDPE/Deca-BDEs. As shown in Figure 3, the BTBPE/Octa-BDEs and DBDPE/Deca-BDEs varied from 0.12 to 60 (AV ± SD: 5.16 ± 12) and from 0.03 to 0.49 (0.12 ± 0.10), respectively. Obviously, the ratios of DBDPE/Deca-BDEs were much lower than those of BTBPE/Octa-BDEs in sediment cores, which might be caused by the significantly higher concentrations of Deca-BDEs (range: 35.2~967,417 ng/g dw) than Octa-BDEs (range: 4.18~3000 ng/g dw). The ratios of DBDPE/Deca-BDEs in the current study were much lower than those reported in non-e-waste recycling sites, such as in sediment from the Dalian Bay (mean: 3.1) and Sishili Bay (9.2) in China [37], and a highly industrialized bay in Korea (range: 0.84~28) [38]. In addition, the ratios of both BTBPE/Octa-BDEs and DBDPE/Deca-BDEs had no clear increase trends with the sediment depth decreasing (Figure 3). However, the increase in DBDPE/Deca-BDEs with time was reported in dated sediment cores from a highly industrialized saltwater lake in Korea [23]. These results suggested that the replacement of traditional BFRs with NBFRs was hysteretic relative to those in industrial and manufacturing areas and that traditional BFRs are still predominant pollutants in e-waste recycling sites.

**Figure 4:**
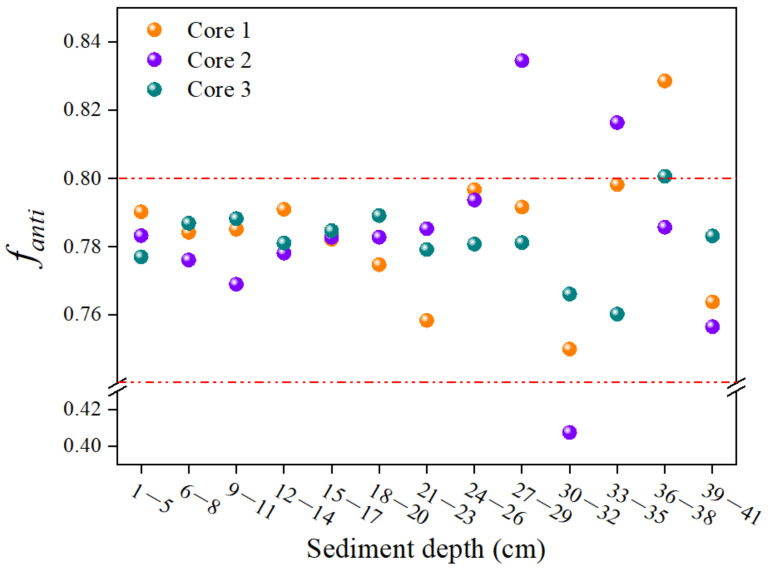
The *f_anti_* values of DP in sediment cores.

The commercial product of DP is primarily a mixture of two stereoisomers, *anti*-DP and *syn*-DP, with the *f_anti_* values (the concentration ratios of *anti*-DP to the sum of *anti*-DP and *syn*-DP) theoretically stabilizing around 0.75, but varying from 0.60 to 0.80 due to variations in product source and batch [39]. Additionally, it may also contain minor byproducts (e.g., *anti*-Cl_11_-DP and *anti*-Cl_10_-DP). In the current study, the concentrations of *anti*-Cl_11_-DP and *anti*-Cl_10_-DP were 27.61 ± 41.62 and 0.63 ± 0.94 ng/g dw in core 1, 17.26 ± 28.60 and 0.63 ± 1.24 ng/g dw in core 2, and 111 ± 159 and 5.01 ± 7.79 ng/g dw in core 3, respectively (Table 1). The total contribution proportions of *anti*-Cl_11_-DP and *anti*-Cl_10_-DP were lower than 1% in all depth layers of the three sediment cores and showed no significant correlations with sediment depth (*Spearman*: *r* = −0.35~0.25, *p* > 0.05), suggesting that *anti*-Cl_11_-DP and *anti*-Cl_10_-DP were mainly contributed from the commercial products of DP rather than degradation after deposition. Additionally, the *f_anti_* value is often used to explore the migration and transformation process of DP in the environment [4]. The *f_anti_* values varied within the ranges of 0.75~0.83, 0.41~0.83, and 0.76~0.80 in sediment cores 1, 2, and 3, respectively, with the corresponding average *f_anti_* values of 0.78 ± 0.02, 0.76 ± 0.11, and 0.78 ± 0.01 (Figure 4). These *f_anti_* values almost fell in the range of 0.60~0.80 (the *f_anti_* of DP technical products) and were in line with those in sediments from Qiantang River (0.75) [40], the Pearl River Delta (0.76 ± 0.04) [41], Lake Taihu (0.75 ± 0.04) [42], and Songhua River (0.77 ± 0.06) [43]. In addition, the variations in *f_anti_* in sediment cores were not significantly related to the sediment depth (*Spearman*: *r* = −0.23~0.04, *p* > 0.05), which also suggested that DP degradation did not occur in sediment cores.

### 3.3. Environmental Burdens of NBFRs and DP

The mass inventory of NBFRs and DP in sediments was calculated as follows (Equation (1)):

(1) $$Inventory=\sum C_{i}\cdot d_{i}\cdot \rho_{i}$$

where *C_i_* (ng/g dw) is the mean concentration of pollutants in sediment layer *i* with its thickness of *d_i_* (cm); and *ρ_i_* (g/cm^3^) is the mass density of the dry sediments, which was assumed to be 1.5 g/cm^3^ [8]. Therefore, these environmental burdens of NBFRs and DP are the total mass of these pollutants from the bottom to the top layers of sediment cores per unit area. The inventories of DBDPE, BTBPE, HBB, PBT, PBEB, *p*TBX, and DP in sediment cores were estimated to be 680,253 ± 674,407, 113,496 ± 109,942, 201,133 ± 320,848, 457 ± 602, 370 ± 641, 113 ± 116, and 696,619 ± 837,387 ng/cm^2^, respectively, which were much higher than those in the sediments of the Pearl River Estuary (DBDPE: 245 ng/cm^2^, BTBPE: 4.10 ng/cm^2^ [44], the e-waste recycling sites in Taizhou (DBDPE: 4110 ng/cm^2^, BTBPE: 960 ng/cm^2^, HBB 690 ng/cm^2^) [8], and a highly industrialized saltwater lake in Korea (DBDPE: 5.4 ng/cm^2^, BTBPE: 40 ng/cm^2^, DP 3.4 ng/cm^2^) [23]. Additionally, it is worth noting that the mass inventories of NBFRs and DP in the current study were calculated using the concentrations in sediment cores rather than surface sediments, which could provide much more realistic stocks, because the concentrations of NBFRs and DP in the bottom layers were much lower than those in the top layers. Based on the average environmental burdens of NBFRs and DP and the area of 5000 m^2^ for the pond, the total environmental burdens of DBDPE, BTBPE, HBB, PBT, PBEB, *p*TBX, and DP were estimated to be 34.0, 5.67, 10.1, 0.02, 0.02, 0.01, and 34.8 kg, respectively.

## 4. Conclusions

In summary, most of the studied NBFR and DP compounds, except a few, such as PBEB, *p*TBX, *anti*-Cl_11_-DP, and *anti*-Cl_10_-DP, were detected in all depth layers of the sediment cores, implying the long-term contamination of NBFRs and DP in the e-waste-polluted pond of an e-waste recycling site in South China. The concentrations of NBFRs (5.71~180,895 ng/g dw) and DP (4.95~109,847 ng/g dw) were at the high end of the global range and showed an increasing trend from the bottom to the top layers of the sediment cores, which could be caused by the increase in the amount of e-waste produced across the world. DBDPE (contribution proportions: 58 ± 15%~73 ± 15%) was the most abundant NBFR, followed by BTBPE (12 ± 15%~34 ± 15%) and HBB (7 ± 6%~17 ± 10%), indicating the extensive use of these three NBFRs around the world. Moreover, the concentration ratios of BTBPE/Octa-BDEs and DBDPE/Deca-BDEs had no clear increase trends with the decrease in sediment depth, suggesting that the replacement of traditional BFRs with NBFRs was hysteretic in e-waste recycling sites. The *f_anti_* values of DP in the sediment cores (0.41~0.83) almost fell in the range of those in DP technical products and were not significantly related to the sediment depths, demonstrating that DP did not undergo degradation in sediment cores. In view of the high concentrations of NBFRs and DP in the studied sediments and the worldwide distribution of e-waste recycling sites, it is urgent to pay more attention to the concentration trends of these contaminants in the soil, atmosphere, organisms, and even human bodies in these hot spots in future studies to assess their ecological risks more scientifically.

## Figures and Tables

**Table 1:** Concentrations (ng/g dw; range and mean ± standard deviation [AV ± SD]) and detection frequencies (DFs) of NBFRs and DP in sediment cores from an e-waste-polluted pond located in a typical e-waste recycling site in South China.

	Sediment Core 1			Sediment Core 2			Sediment Core 3		
	DF	Range	AV ± SD	DF	Range	AV ± SD	DF	Range	AV ± SD
*p*TBX	23%	n.d. ^a^~3.24	0.59 ± 1.15	23%	n.d.~5.6	0.79 ± 1.69	62%	0~32.9	4.06 ± 8.89
PBT	100%	0.03~8.07	1.18 ± 2.25	100%	0.04~9.62	1.64 ± 3.03	100%	0.29~64.0	16.4 ± 24.2
PBEB	23%	n.d.~0.03	0 ± 0.01	46%	n.d.~89.5	14.4 ± 29.3	0%	n.d.	n.d.
HBB	100%	0.35~1202	184 ± 371	100%	0.41~1135	239 ± 416	100%	73.9~39,130	7764 ± 13,604
BTBPE	100%	0.93~4750	886 ± 1523	100%	1.44~3087	520 ± 944	100%	17.7~12,678	3450 ± 4229
DBDPE	100%	4.41~8014	1383 ± 2719	100%	7.58~56,947	5855 ± 15,724	100%	664~128,990	22,136 ± 37,000
∑_6_NBFRs	100%	5.71~13,977	2455 ± 4617	100%	9.47~61,273	6631 ± 17,118	100%	756~180,895	33,371 ± 54,866
*syn*-DP	100%	1.16~3880	957 ± 1400	100%	1.8~1882	418 ± 654	100%	69.6~24,386	4912 ± 8262
*anti*-DP	100%	3.74~14,179	3520 ± 5140	100%	6.6~6800	1463 ± 2317	100%	228~84,979	17,704 ± 29,573
*anti*-Cl_10_-DP	69%	n.d.~2.56	0.63 ± 0.94	62%	n.d.~4.29	0.63 ± 1.24	62%	0.01~22.6	5.01 ± 7.79
*anti*-Cl_11_-DP	100%	0.02~111	27.6 ± 41.6	100%	n.d.~92.1	17.26 ± 28.6	100%	1.63~459	111 ± 159
∑_4_DP	100%	4.95~18,169	4506 ± 6582	100%	8.4~8779	1899 ± 3001	100%	299~109,847	22,732 ± 37,997

^a^: not detected.

## Data Availability

Data are contained within the article.
